# Electrospun PA6 Nanofibers Bearing the CeO_2_ Dephosphorylation Catalyst

**DOI:** 10.1021/acsomega.3c03561

**Published:** 2023-07-12

**Authors:** Jiří Henych, Petr Ryšánek, Martin Št’astný, Zuzana Němečková, Slavomír Adamec, Martin Kormunda, Simona Kamínková, Kateřina Hamalová, Jakub Tolasz, Pavel Janoš

**Affiliations:** † Institute of Inorganic Chemistry of the Czech Academy of Sciences, Husinec-Řež 250 68, Czechia; ‡ Faculty of Environment, Jan Evangelista Purkyně University in Ústí nad Labem, Pasteurova 3632/15, Ústí nad Labem 400 96, Czechia; § Faculty of Science, Jan Evangelista Purkyně University in Ústí nad Labem, Pasteurova 3632/15, Ústí nad Labem 400 96, Czechia

## Abstract

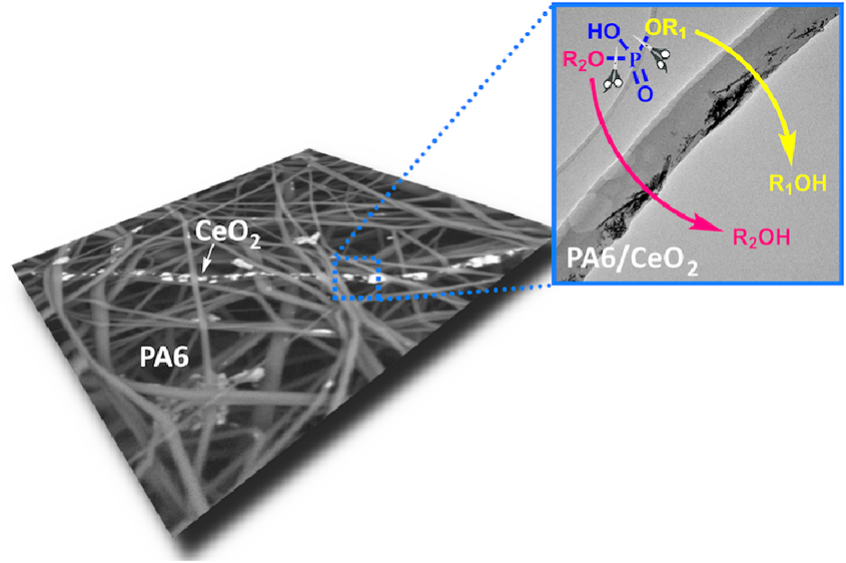

Two types of CeO_2_ nanoparticles (CeNPs) prepared by low-temperature (<100 °C) precipitation methods in water were successfully immobilized in a matrix of electrospun PA6 nanofibers. The colloidal solutions of CeNPs in AcOH were directly mixed with the polymer solution before the needle electrospinning process, thereby achieving their good dispersion in the nanofibers. CeNPs embedded in the structure and on the surface of nanofibers exposing their reactive surfaces showed robust dephosphorylation catalytic activity, as demonstrated by monitoring the hydrolytic cleavage of three phosphodiester molecules (*p*-NP-TMP, *p*-NPPC, BNPP) in water by the HPLC method. This procedure allowed us to study the kinetics and mechanism of the hydrolytic cleavage and the ability of immobilized CeNPs to cleave different types of P–O bonds. One of the main hydrolysis products, *p*-nitrophenol, was effectively adsorbed on PA6 nanofibers, which may allow the selective separation of the degradation products after hydrolysis.

## Introduction

The energy of cerium 4f orbitals is very close to valence 5d^1^ and 6s^2^ orbitals, which endows cerium with dual valence states and very characteristic physicochemical properties.^1^ Cerium occurs naturally in oxides such as cerium dioxide (ceria), a typical representative of reducible oxides,^2^ which require low energy for oxygen vacancy formation, and the electrons left after reduction are localized in the cations, decreasing their oxidation state and presenting open-shell electronic states. The easy formation of oxygen defects^3^ and high oxygen storage capacity and mobility, together with the relatively stable cubic fluorite structure,^4^ give CeO_2_ extraordinary redox^5^ and acid–base^6^ properties. Therefore, ceria is used in many conventional applications^4^ from glass polishing to three-way catalysts. However, its potential goes much further, especially with ceria nanoparticles (CeNPs or nanoceria), which are intensively studied for applications in many catalytic reactions,^4^ sensors,^7^ optics,^8^ or solid oxide fuel cells,^9^ or even in biotechnology, environmental sciences,^10^ and biomedicine.^11,12^

Nanoceria is becoming very popular due to its exceptional multi-enzyme mimicking properties.^11^ Although several inorganic nanomaterials have been introduced as enzyme mimics (so-called nanozymes),^13^ it is difficult to find any more versatile than nanoceria, which have oxidase,^14^ peroxidase,^14−16^ catalase,^14,17^ superoxide dismutase,^15,17^ or phosphatase^18^ mimetic activities. In a series of our recent works, we described the dephosphorylation capabilities of prepared and commercial nanoceria on ATP-like substrates^19^ and other biologically relevant nucleotides such as nicotine adenine dinucleotide (NAD) and thiamine pyrophosphate (TPP),^20^ or even more resistant phosphoester bonds, specifically those in the 3′,5′-cyclic adenosine monophosphate (cAMP),^21^ which mimics bonds in nucleic acids. Recently, we developed an analytical procedure to follow the hydrolytic cleavage of *p*-nitrophenyl phosphorylcholine (*p*-NPPC) and *p*-nitrophenyl thymidine 5′-monophosphate (*p*-NP-TMP),^22^ allowing to distinguish selective cleavage of certain phosphoester bonds, i.e., phospholipase C- and D-like activity. Moreover, CeNPs prepared by very simple low-temperature water-based precipitation methods^22^ had high surface area (>150 m^2^/g), good colloidal stability, and attractive antiviral properties against both enveloped (HSV1) and non-enveloped (Ad5) viruses. Immobilization of loose CeNPs on surfaces including fibers would be desirable to prevent their aggregation and to control their release into the media or the environment.

Nanoscale polymeric fibers are promising materials with a wide range of intended or already commercial applications including filtration membranes,^23,24^ electronics,^25^ or ion batteries,^26^ and they are especially attractive for biomedical applications.^27^ Several natural polymers are very similar or almost identical to macromolecular compounds in the human body, which makes them ideal for example for scaffolding and tissue engineering.^28^ Nevertheless, functionalization of nanofibers may significantly extend their area of applications. Nylon 6 (PA6) is a synthetic polymer with high strength, good chemical resistance, and water tolerance, and PA6 fibers are currently the second most produced synthetic fibers in the world.^29^ PA6 nanofibers are therefore an excellent candidate for further modification/functionalization by various materials and techniques, either for improving nanofiber properties^30,31^ or for specific applications such as sensing,^32^ antiviral mask filters,^33^ batteries,^34^ or bone regeneration.^35^

Anchoring enzymes onto the fibers may improve enzyme operational stability, reusability, and product separation making 1D fibrous materials one of the most desirable supports for enzyme immobilization, as elaborated in several review articles.^36,37^ However, the works on immobilization of inorganic nanoparticles with enzyme-like catalytic activity are relatively scarce. Hu et al. prepared PVA nanofibers with CeO_2–*x*_ nanorods showing haloperoxidase-like activity giving the fibers antibiofouling properties,^38^ and PVA/hematite composites with catalase-like activity for better wound healing.^39^ PAN nanofibers decorated with Fe nanoparticles having oxidase and peroxidase-like activities were also used against bacteria (*Escherichia coli* and *Staphylococcus aureus*) and for better wound healing.^40^ However, to the best of our knowledge, the nanofibers carrying inorganic nanoparticles with dephosphorylation catalytic properties have not been yet reported.

Herein, the PA6 electrospun nanofiber networks carrying functional CeO_2_ nanoparticles with dephosphorylation catalytic activity were prepared. Two types of CeNPs were prepared by simple low-temperature water-based precipitation methods without autoclaving or any calcination, and their AcOH colloidal solutions were easily mixed with PA6 polymer/acetic–formic acid solution to achieve perfect dispersion of CeNPs within the electrospun fibers. As demonstrated, CeNPs were successfully embedded in the structure and onto the surface of the PA6 fibers and retained their pseudo-enzymatic (in this case dephosphorylation) activity as tested by hydrolytic cleavage of three different organophosphate diesters.

## Materials and Methods

### Nanoceria Synthesis

CeNPs were prepared by two low-temperature (<100 °C) precipitation synthesis procedures without any calcination reported elsewhere.^22^ In short, the CeAMM sample was prepared by precipitation of cerium(III) nitrate hexahydrate water solution with ammonium hydroxide under CO_2_-free ambient air followed by aging at 60 °C. The CePER sample was prepared by precipitation of cerium(III) hydroxide in water followed by oxidation with H_2_O_2_ and refluxing at water boiling temperature. The prepared CeNPs in water were washed by decantation, and the solvent was replaced to pure AcOH (to obtain suspension with ceria/AcOH concentration 1 mg/mL) for further use.

### Synthesis of PA6 Nanofibers and Electrospinning Conditions

Nylon 6 (PA6) Ultramid B24 granules were purchased from BASF company (Germany). PA6 was dissolved in 2:1 solution of acetic and formic acids by gentle stirring for 15 h at 45 °C to prepare 16 wt % solution. For PA6 modified with CeNPs, the colloidal solutions of CeAMM and CePER were added directly to the PA6 polymer solution in concentration 1 wt %. The prepared spinning solution was immersed in an ultrasonic bath for 1 h for perfect dispersion of CeO_2_ nanoparticles in the polymer solution. The electrospinning process was carried out on a commercial InoSPIN mini device (InoCURE, Czech Republic) using the needle spinning process. The condition in the spinning chamber were set to 25 °C and 40% relative humidity. An 18 G needle was used with a polymer solution dosing rate of 0.1 mL/min, and the amount of solution per sample was 4 mL. The voltage applied to the spinning electrode was 45 kV, and the voltage applied to the collecting electrode (rotation cylinder) was −10 kV. The nanofibers were collected on the polypropylene spunbond fabric.

### Characterization Methods

A PANalytical X’Pert PRO diffractometer with Cu Kα radiation (<λ> = 1.5418 Å) was employed to collect X-ray diffraction patterns. A Rigaku Primus IV WDXRF spectrometer was used for X-ray fluorescence (XRF) measurements using SQX software with a standardless method of fundamental parameters. X-ray photoelectron spectroscopy (XPS) was used to investigate the surface chemistry using a PHOIBOS 100 spectrometer (Specs, Germany) operating in fixed analyzer transmission mode and medium area settings with an Al X-ray tube (Kα line with energy 1486.6 eV) and 40 and 10 eV pass energies to obtain a survey and high-resolution spectra, respectively; the measured area is about 1.4 × 4 mm^2^. CasaXPS software was used for data processing using a Shirley background profile. Scanning and transmission electron microscopes (SEM and TEM) were used to study the morphology, structure, and elemental composition on the FEI Nova NanoSEM 450 and FEI Talos F200X microscopes (both Thermo Fisher Scientific) with TEM/EDS elemental mapping. For ICP-MS analysis, samples of nanofibers on non-woven fabric were cut into rectangles of approx. 2 × 3 cm (three replicates for each sample with an average weight of 0.015 g ± 0.001) and dissolved in a mixture of 6 mL of HCl (35% p.a.) and 2 mL of HNO_3_ (65% p.a.) in the Anton Paar Multiwave 5000 microwave device. The temperature of the decomposition mixture increased at a rate of approximately 10–15 °C/min to (175 ± 5) °C and was maintained at this temperature for (10 ± 1) min until complete decomposition of the nanofibrous fabric. After cooling, deionized water was added to the solutions to a volume of 10 mL and mixed and then diluted 10 times and analyzed on an ICP-MS (Agilent 7900 hyperbolic quadrupole system with an ORS 4 collision reaction cell and an orthogonal detector system). A tuning calibration solution (10 ppb Li, Co, Y, Ce, Tl) and a 100 ppb indium internal standard with a four-step calibration series (0.01; 0.1; 0.5; 1 and 2 mg/L in 2% HNO_3_ from the standard Astasol AN9088MN) were used. The mineralization blank (acids without sample) was subtracted from the measured concentration, and the resulting concentrations were converted to mg/kg. The surface charge, chemistry, and polarity of nanofibers were determined using electrokinetic analysis. The electrokinetic potential (zeta potential) of the tested surfaces was measured on a SurPASS instrument (Anton Paar, Austria). For each measurement, a pair of samples with the same surface was mounted on two sample holders (with a cross section 2 × 1 cm^2^). The measurements were performed inside an adjustable gap cell (with a gap of 100 μm) in contact with an electrolyte (0.001 mol·dm^–3^ KCl) at room temperature and constant pH = ∼6. The streaming current method was used, and the Helmholtz–Smoluchowski equation was applied to calculate the zeta potential. All samples have been measured four times with an experimental error of 5%. A Thermo Nicolet NEXUS 670 FTIR spectrometer equipped with an MCT detector and ZnSe ATR crystal was used to collect IR spectra (obtained by accumulating 128 scans with a resolution of 4 cm^–1^), while a DXR Raman confocal microscope with a 532 nm excitation laser was used to record Raman spectra. The DFT-specific surface area and pore volume were determined by nitrogen (Linde, 99.999% purity) adsorption/desorption isotherms using Quantachrome Instruments NOVAtouch LX2 (Anton Paar, Austria). The nanofibrous samples were outgassed at a laboratory temperature (25 °C) for 20 h before measurement.

### Dephosphorylation Catalytic Activity

The dephosphorylation activity was determined by following ceria-catalyzed hydrolysis of three different molecules, *p*-NP-TMP, *p*-NPPC, and bis(*p*-nitrophenyl)phosphate (BNPP), by the HPLC/DAD method. An analytical procedure developed previously^22^ was used to follow the kinetics of hydrolysis and to identify and quantify the hydrolysis products: *p*-nitrophenol (*p*-NP), thymidine, and choline. The measurement procedure is described in detail in the Supporting Information (SI).

## Results and Discussion

### Characterization of CeNPs

**Figure 1:**
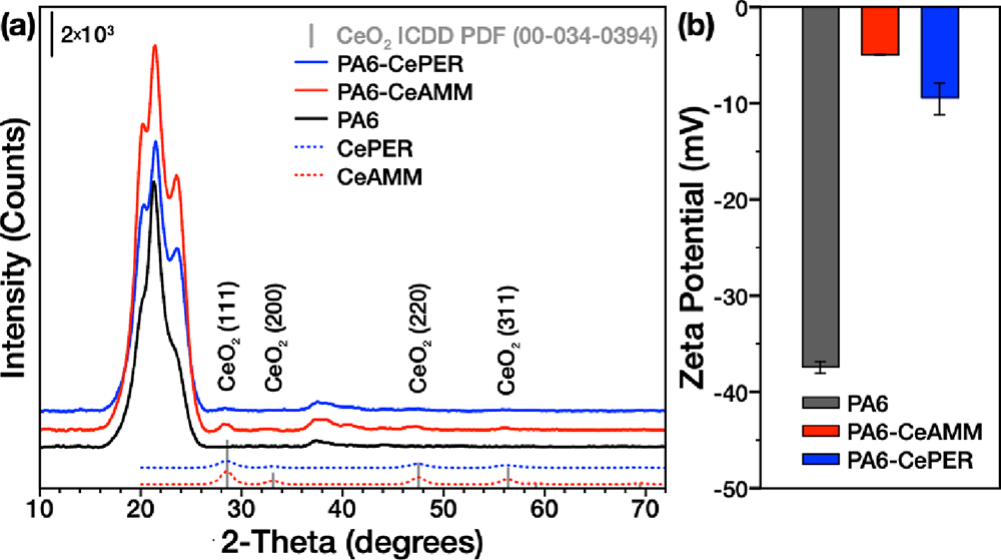
(a) XRD patterns of pristine PA6 nanofibers and CeO_2_ nanoparticles (CeAMM and CePER), and PA6 fibers bearing CeO_2_ nanoparticles (PA6-CeAMM, PA6-CePER). (b) Zeta potentials of the prepared nanofibers.

The CeNPs with high dephosphorylation catalytic activity were prepared by two simple precipitation methods reported previously.^22^ The CeNPs were thoroughly described by numerous methods in a previous study^22^ and show some similarities but also distinct features depending on the synthesis. The diffractograms of both samples (Figure 1) show the typical diffraction lines of the face-centered cubic CeO_2_ structure (ICDD PDF 00-034-0394), with the calculated crystallite sizes (using the Scherrer method) of 3.8 nm (CeAMM) and 2.9 nm (CePER). The small crystallite size also corresponds to the relatively large specific surface area obtained by nitrogen physisorption reaching ∼150 and 180 m^2^/g for CeAMM and CePER, respectively.^22^ The different nanostructure morphology of mostly spherical (CeAMM) and irregular-shaped particles with sharp edges and corners (CePER) can be seen on HRTEM images (Figure S1) in the SI. In addition, in the CeAMM sample, the formation of CeO_2_ nanorods consisting of primary spherical particles was also observed (Figure S1).

### Characterization of Pristine and CeNP-Modified PA6 Nanofibers

AcOH colloidal solutions of CePER and CeAMM were mixed directly with polymer PA6 solution in 2:1 acetic/formic acid before electrospinning to integrate CeNPs into the nanofiber structure. XRD analysis of the pristine and CeNP-modified PA6 nanofibers shows a characteristic diffraction pattern between 16 and 26° 2θ, indicating the presence of both α and γ phases of PA6, consistent with previous findings.^41,42^ Interestingly, the modification of PA6 fibers with CeNPs does not lead to smoothing and broadening of the reflection profiles due to the restriction of PA6 crystallization, which leads to the formation of smaller crystalline domains in the modified fibers, as observed previously in the case of modification of PA6 nanofibers with DTAB, BTAB, or CHX antibacterial agents.^41,42^ On the contrary, both types of CeNPs in the nanofibers promote PA6 crystallization, as evident from the highly intense and sharper diffraction lines. Furthermore, diffraction lines related to CeO_2_ were clearly visible in PA6 fibers modified with CeAMM, while only the most intense diffraction line belonging to the (111) planes of CeO_2_ can be recognized in PA6-CePER. This can be caused by the different dispersion of CeNPs prepared by distinct methods within the nanofibers. Moreover, from the XRD patterns of bare CeNPs, it is evident that the CeAMM sample has larger crystallites and thus higher diffraction line intensities compared to the CePER sample.

Elemental composition of the prepared samples obtained by XRF analysis (Table 1) shows that pure PA6 nanofibers contain some small traces of P, Cl, and K. Upon modification with CeNPs, in addition to the same residues (and Si), 0.22 and 0.18 wt % of Ce were determined in PA6-CeAMM and PA6-CePER, respectively. The amount of Ce in the samples was also determined by ICP-MS after the complete decomposition of the nanofibers in aqua regia and was found to be 4.25 ± 0.06 (PA6-CeAMM) and 3.70 ± 0.75 (PA6-CePER) mg/g. Both methods show a slightly lower amount of Ce in PA6-CePER and the value variance for three independent measurements (ICP-MS method) expressed as standard deviation that was noticeably higher for this sample. This suggests less homogeneous distribution of CeNPs within the PA6-CePER fibers, which may be caused by stronger nanoparticle agglomeration or the formation of rigid aggregates in the AcOH solution used for mixing with polymer solution before electrospinning. Nevertheless, for both PA6-modified samples, the nanofibers demonstrably contained CeO_2_ particles. The XPS spectra (Figures S2 and S3) of the pure and modified fibers were almost identical. Due to the small penetration depth of the XPS measurement and the structure of the fiber samples, it was not possible to confirm and determine the amount of Ce in the fibers. Nevertheless, from the high-resolution spectra (Figure S3), hints of the peak positions of the Ce3d components in the random noise of the Ce_AMM sample can be seen.

**Table 1:** Relative Chemical Composition Obtained by XRF Analysis

component	PA6, wt %	PA6-CeAMM, wt %	PA6-CePER, wt %
Si		0.016	0.065
P	0.073	0.066	0.053
Cl	0.024	0.020	0.039
K	0.070	0.083	0.084
Ce		0.218	0.176
PA6 (calc.)	99.8	99.6	99.6

**Figure 2:**
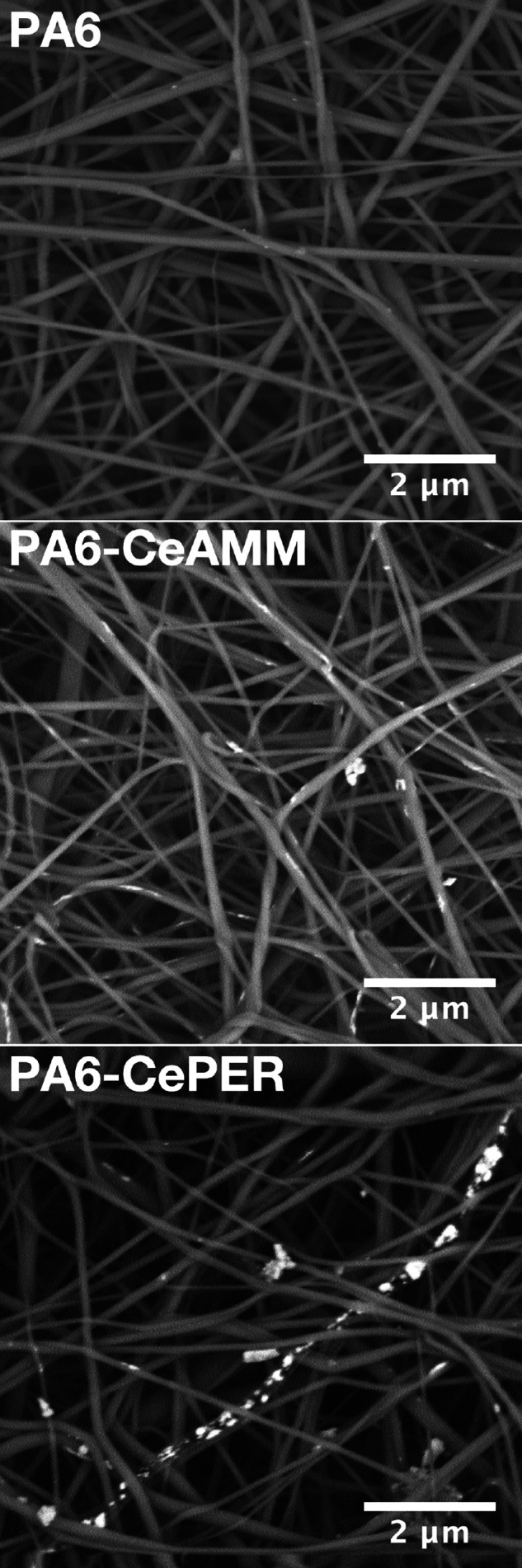
SEM images of pristine and CeO_2_-modified PA6 nanofibers.

**Figure 3:**
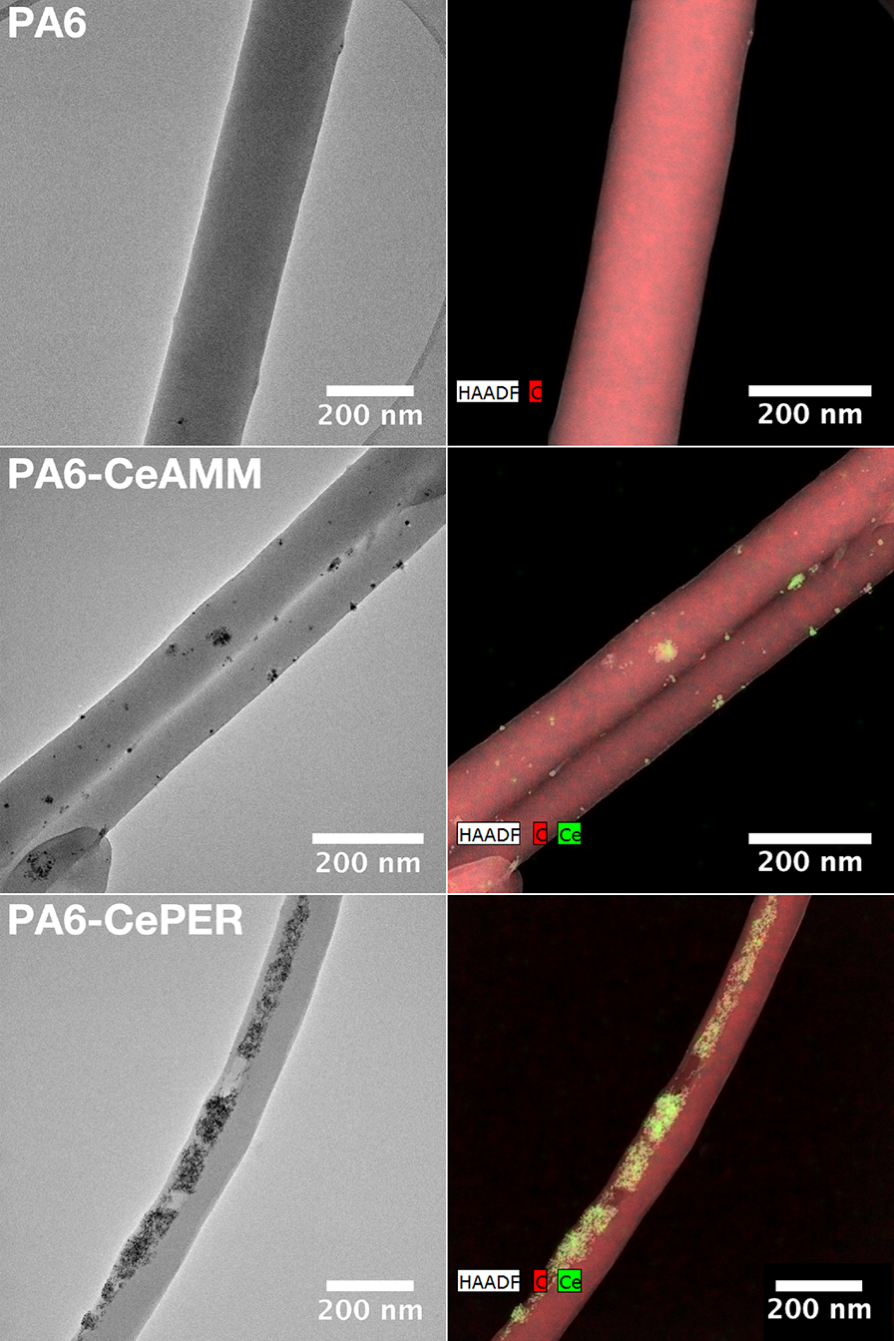
TEM images of pristine and CeO_2_-modified PA6 nanofibers (left) with EDS elemental mapping (right).

**Figure 4:**
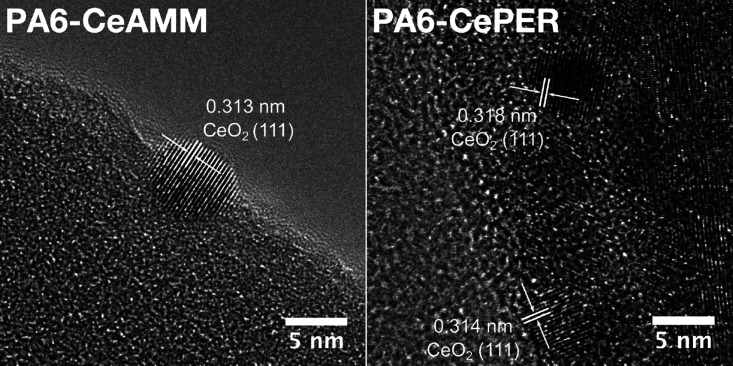
HRTEM images of CeO_2_ nanoparticles on PA6 nanofibers.

The morphology, structure, and distribution of CeNPs within the PA6 nanofibers were studied by both SEM (Figure 2) and HRTEM (Figures 3 and 4) with EDS mapping. SEM analysis was performed using a circular backscatter (CBS) detector that enhances the material contrast so the parts of the sample consisting of heavier elements have a much stronger signal and are shown as significantly brighter. As can be seen, in all three samples, individual fibers with a mainly spherical cross section with slightly variable thicknesses are often stuck together to form a dense nanofibrous network with frequent crossings, knots, and twists. The morphology, shape, or thickness of the nanofibers did not change significantly upon CeNP modification, indicating relatively good dispersion of CeNPs in polymer solution before and during the electrospinning process. Due to the enhanced material contrast, the CeNPs are clearly visible in modified fibers. The CeNPs or their nanosized aggregates are mainly distributed inside the fibers (especially for the PA6-CePER sample) and also on their surfaces. Some aggregated particles also appear to be attached to the fibers from the outside, presumably by electrostatic forces (discussed below).

TEM analysis (Figure 3) of the modified fibers shows clearly that nanoparticles were successfully immobilized in or onto PA6 nanofibers. Their distribution within the fibers is slightly different for two types of CeNPs used. In PA6-CeAMM, individual particles are very well dispersed throughout the body of the nanofibers, while in the PA6-CePER sample, denser aggregates are formed and distributed inside and near the surface of the fibers. EDS mapping (Figure 3, right column) confirmed cerium as the main element in the nanoparticles. HRTEM analysis (Figure 4) was performed to further study the crystalline structure and morphology of the individual particles. As evident, individual CeNPs were successfully embedded in the structure of the nanofibers, but they are also exposed and thus available for any surface reaction. Both the electron diffraction analysis (Figure S4) and identified d-spacing (0.31 nm) assigned to (111) planes of CeO_2_ confirmed a presence of CeNPs with a preserved highly crystalline structure in modified PA6 samples. Therefore, it can be concluded that the use of colloidal solutions of CeNPs directly in the polymer solution before electrospinning is a good strategy to prepare CeO_2_-modified fibers without compromising the quality of the original fibers. Some additional TEM images of fibers at lower magnification can be seen in Figure S5.

Electrokinetic analysis can determine the changes in surface charge and chemistry induced by nanofiber modification (Figure1b, Table S1). The zeta potential of pure PA6 nanofibers is −37.40 ± 0.60 mV, fairly consistent with previously obtained values.^41,43^ On the contrary, as investigated previously,^22^ the zeta potentials of CeAMM and CePER nanoparticles are highly positive (ca. 20.1 ± 6.8 mV for CeAMM and 30.6 ± 7.6 mV for CePER, at pH = 5.5). The zeta potential values over 30 mV would lead to good colloidal stability of the nanoparticles in solution,^44^ which is important for preparing stable polymer/CeNP solution. Furthermore, the positive zeta potential of CeNPs is beneficial for their efficient adhesion to the negatively charged PA6 during the electrospinning process. Indeed, in both CeNP-modified nanofibers, the zeta potential significantly decreased to −4.95 ± 0.1 (PA6-CeAMM) and −9.41 ± 1.61 mV (PA6-CePER) after modification. This change in surface charge also suggests that the CeNPs were deposited on the surface or protrude from the interior of the nanofibers during electrospinning and, importantly, their surfaces are exposed, which is consistent with HRTEM analysis. The change in zeta potential was more significant for the PA6-CeAMM sample, despite the fact that pure CeAMM has a lower positive zeta potential than CePER, suggesting more CeNPs with exposed surfaces in this sample.

Vibrational spectroscopies were used to further study structural properties of the nanofibrous mats (Figure S6). Both Raman and FTIR spectra show characteristic vibrations of nylon 6 fibers.^45^ The spectra of pristine PA6 fibers and upon modification are practically identical, which indicates that the structure of fibers was not disturbed upon modification with CeNPs.

The DFT specific surface area (SSA) obtained by low-temperature nitrogen physisorption (Table S2) did not show significant changes upon modification of pristine PA6 fibers. The pristine PA6 nanofibrous mat had an SSA 10.5 ± 2.6 m^2^/g, while values 11.4 ± 0.2 and 7.0 ± 0.5 m^2^/g were found for PA6-CeAMM and PA6-CePER, respectively.

It follows from the material characterization that CeNPs were successfully embedded into PA6 fibers (or onto their surfaces) by a simple and gentle method, without losing the quality of the original fibers.

### Dephosphorylation Catalytic Activity of PA6 Nanocomposite Mats

**Scheme 1:**
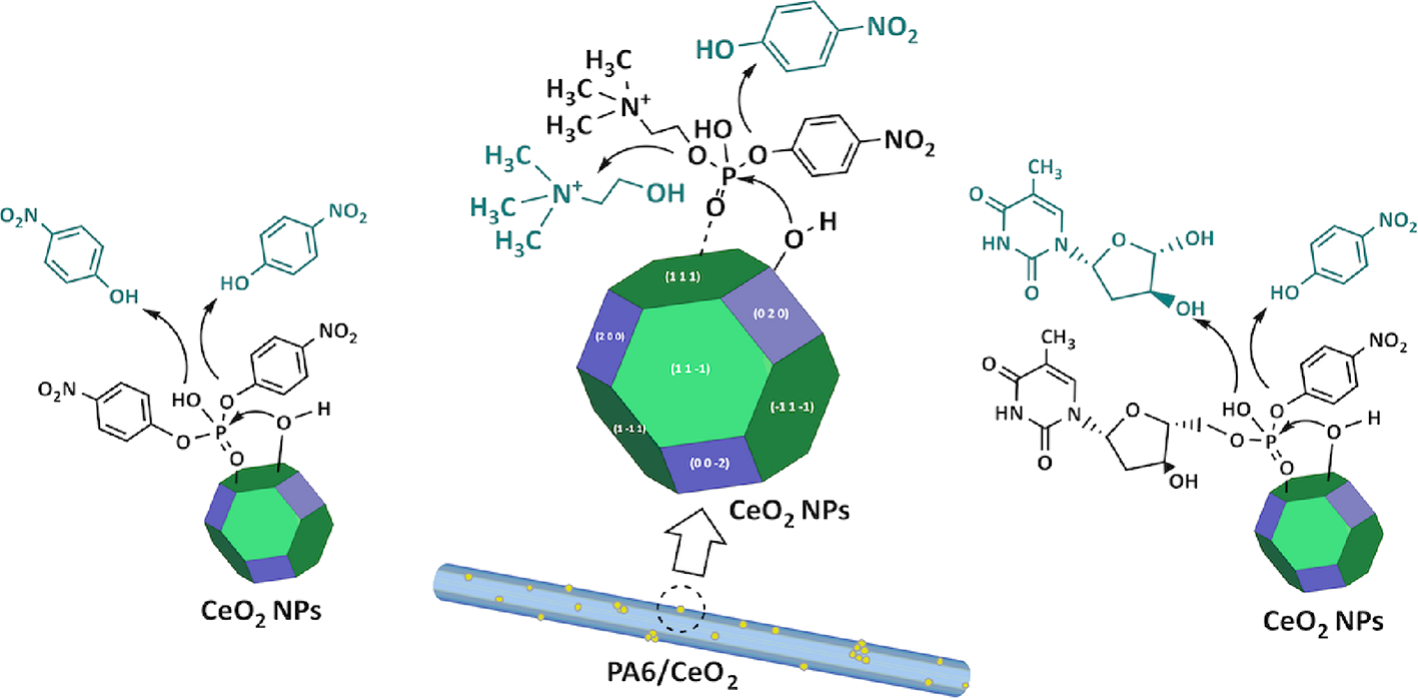
Possible Mechanism of Hydrolytic Cleavage of Three Phosphodiesters—BNPP (Left), *p*-NPPC (Center), and *p*-NP-TMP (Right) on the Surface of PA6-Anchored CeNPs

To prove the successful immobilization of the functional CeNPs in nanofibrous networks, the dephosphorylation catalytic properties were evaluated by the method developed previously^22^ for testing of pristine CeNPs. Three different phosphate diester molecules were used to study catalytic activity following both the degradation of the parent compound and the generation of its degradation products by the HPLC method. Concretely, *p-*NPPC is a chromogenic substrate that is used to measure phospholipase C (PLC) activity, while *p-*NP-TMP is a classical nucleotide phosphodiesterase substrate similarly to bis(*p*-nitrophenyl) phosphate (BNPP), which is also often used for estimation of phosphodiesterase (PED) activity.^46^ Thanks to the analytical procedure developed previously,^22^ we were also able to distinguish cleavage of different P–O bonds in *p-*NPPC and *p-*NP-TMP substrates, thus discerning the phospholipase C- and D-like activity of CeO_2_-based nanomaterials. The proposed mechanism of hydrolytic decomposition of all three substrates on the surface of CeNPs is presented in Scheme 1.

It is widely accepted that the hydrolytic decomposition of phosphoesters on the surface of CeNPs proceeds via the S_N_2 nucleophilic substitution mechanism. The coordination of the phosphoryl oxygen of the phosphoester molecule to the Ce^4+^ cation (Lewis acidic site) aids the polarization of the P=O bond, which leads to the charge accumulation in the oxygen atom and the activation of the P central atom. Subsequently, the surface hydroxyl group bound to the Ce^3+^ cation (nucleophile) attacks the P atom with expulsion of the leaving group. Diesters are the least reactive phosphate esters toward hydrolysis (compared to tri- and monoesters), but the phosphoester hydrolysis rate is also strongly dependent on the basicity of the leaving group and other substituents.^47^ Herein, diesters that are extremely resistant to solvolytic cleavage, making them suitable functionalities for the backbones for DNA and RNA,^48^ are investigated.

The pristine PA6 nanofibers show distinct adsorption of diesters from aqueous solution (conc. = 1 mg/L), reaching 16, 19, and 3% of adsorbed *p*-NP-TMP, *p*-NPPC, and BNPP, respectively, after 240 min (Figure S7). However, the absence of any degradation product indicated that PA6 fibers have no hydrolytic cleavage activity toward any of the three molecules tested. On the other hand, both PA6 mats modified with CeNPs show the reactivity toward all three diesters with the formation of the several degradation products, proving robust PED-like activity inherent to CeNPs.

### The Kinetics of Dephosphorylation Reactions

**Figure 5:**
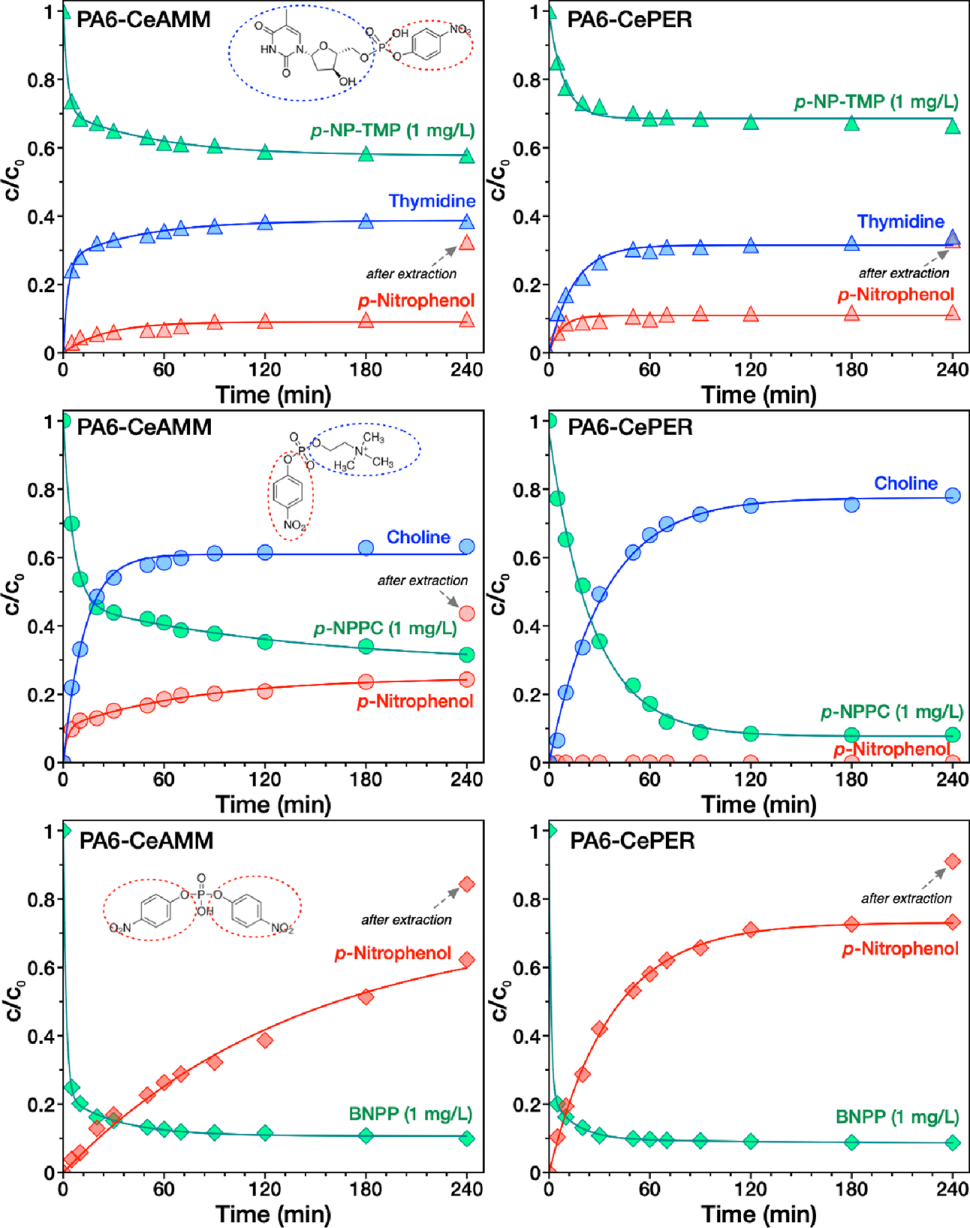
Dephosphorylation catalytic activity of PA6-CeAMM nanofibers (left column), and PA6-CePER (right column) toward *p*-NP-TMP (top line), *p*-NPPC (middle line), and BNPP (bottom line) in water.

In detail (Figure 5), *p*-NP-TMP hydrolysis on the surface of PA6-CeAMM and PA6-CePER nanofibrous mats results in cleavage of both thymidine and *p*-NP, which indicates the ability of nanoceria to break both P–O bonds from the parent molecule. Interestingly, both the rate of increase and the amount of thymidine are higher than those of *p*-NP, which is in contrast to our previous investigation on pristine CeNPs, where we instead observed a slightly faster formation of *p*-NP than thymidine. However, after extraction of PA6-CeAMM and PA6-CePER with methanol:acetonitrile (1:1) solution, we observed that a significant amount of *p*-NP was adsorbed on the nanofibrous mats. Therefore, we have tested the adsorption of pure *p*-NP solutions (0.3, 0.46, 0.8 mg/L, which were equimolar to 1 mol of BNPP, *p*-NPPC, and *p*-NP-TMP) on pristine and CeNP-modified PA6 nanofibrous mats (see Figure S8). As evident, the PA6 nanofibers have strong ability to adsorb *p*-NP from the solution, which explains the lower amount of *p*-NP released to the solution after hydrolysis by CeNPs immobilized in the PA6 matrix. This means that there was no reduction in the activity of CeNPs toward *p*-NP-TMP, which are able to cleave both P–O bonds, but one of the products can be effectively captured on PA6 nanofibers. This may be an interesting property for separation of the products. PA6-CeAMM seems to be more effective than PA6-CePER, suggesting better distribution and higher availability of CeNPs within the PA6 nanofibers, which is coherent with TEM investigation.

Furthermore, both modified PA6 fibers were also efficient in hydrolytic cleavage of *p*-NPPC, but here, a significant difference in activity was observed. PA6-CeAMM was effective in cleavage of choline (PLD-like activity) and also *p*-NP (PLC-like activity), which was, however, again found to be released slower and in lesser amount likely due to its strong adsorption in PA6 nanofibers. The amount of *p*-NP after extraction was still slighter lower than the amount of choline (which was equimolar to *p*-NPPC). On the contrary, in the PA6-CePER sample, only choline as a product of hydrolysis was detected even after extraction procedure, indicating that only a PLD-like activity was observed on this sample. However, interestingly, the rate and the amount of *p*-NPPC decomposed and the choline formed are higher than on the PA6-CeAMM sample. This indicates that the degradation of one of the P–O bonds is preferred over the other, which would allow control of the dephosphorylation activity of immobilized CeNPs with the possibility of selective dephosphorylation of a certain degradation of product, in this case choline. Controlling the selectivity of enzymatic reactions on artificial enzymes is often problematic. However, the mechanism of this selective cleavage is unclear and needs further investigation.

The hydrolytic cleavage of the BNPP molecule giving *p*-NP as a product was also achieved on both PA6-modified samples, suggesting their robust dephosphorylation activity. A significant amount of *p*-NP was again entrapped on the PA6 nanofibers as shown by solvent extraction. Interestingly, the BNPP hydrolysis rate is almost identical on both PA6-CeAMM and PA6-CePER samples, but they differ in the rate of *p*-NP release, which suggest different adsorbate–adsorbent interactions. Nevertheless, the amount of *p*-NP produced after solvent extraction is practically identical for both samples.

Note that the detected amount of hydrolysis products on ceria-modified nanofibers was not fully stoichiometric, which was probably due to irreversible adsorption of the products onto the fibers, or some amount of the starting substrate was only adsorbed onto the fibers without subsequent degradation.

### The Effect of Solvent on Catalytic Activity

It is well known that the solvent has a crucial effect on the dephosphorylation activity. Janoš et al.^49^ investigated the degradation of the model compound parathion methyl in different solvents and their mixtures. The degradation reaction proceeds well in nonpolar solvents as well as in aprotic solvents, while protic solvents, such as water, has a detrimental effect on the efficiency. This is probably due to the solvation and inactivation of the surface −OH groups, which are important in phosphoester degradation. Indeed, we have observed previously^22^ the promoting effect of acetonitrile compared to water on dephosphorylation activity of pristine CeNPs. Therefore, we have investigated the reaction kinetics of all three diesters on PA6-CeNPs in acetonitrile instead of water (Figure S9). Interestingly, contrary to expectations, the activity in acetonitrile deteriorated slightly for all three diesters on both types of CeNP-modified fibers. Furthermore, no *p*-NP was identified for BNPP adsorption, even after extractions, which means that it was not degraded in acetonitrile but only adsorbed on the surface. Evidently, this happened after the immobilization of CeNPs within the PA6 nanofiber matrix, so we tentatively assigned this negative effect to some interaction of the PA6 fibers with acetonitrile solvent. However, importantly, the dephosphorylation catalytic activity was proven in water, which is important for any environmentally or biologically oriented application of the functional CeNPs successfully immobilized within the PA6 nanofibrous matrix.

## Conclusions

Two types of CeNPs prepared by simple precipitation methods were successfully immobilized in the matrix of PA6 nanofibrous mats. Good dispersion of CeNPs in nanofibers was achieved by directly mixing colloidal solutions of CeNPs in the PA6 polymer before the electrospinning process. As shown, CeNPs were embedded into the structure and surface of the nanofibers, exposing their active surfaces without compromising the quality of the PA6 nanofibers. Importantly, the robust catalytic activity of the CeNP-modified PA6 nanofibers in water was demonstrated by dephosphorylation of three different phosphodiester molecules. The ability to cleave different types of P–O bonds (PLC- and PLD-like activity) was shown on *p*-NP-TMP and *p*-NPPC substrates, although one type of CeNPs immobilized on PA6 fibers shows selective hydrolysis of only one P–O bond in the *p*-NPPC substrate. Interestingly, the hydrolysis product *p*-NP is effectively adsorbed on PA6 nanofibers, which could be useful for the selective separation of the degradation products unattainable on bare CeNPs.

## Supporting Information Available

HRTEM images of CeNPs prepared by two different precipitation methods; high-resolution XPS spectra of C 1s, O 1s, and N 1s of pure PA6 nanofibers, PA6-CePER, and PA6-CeAMM; high-resolution XPS spectra of Ce 3d on PA6-CePER and PA6-CeAMM; electron diffraction analysis of the PA6-CeAMM and PA6-CePER samples; TEM images of pristine and CeO2-modified PA6 nanofibers; FTIR and Raman spectra of pristine and CeO_2_-modified PA6 nanofibers; dephosphorylation activity of pure PA6 nanofibers towards *p*-NP-TMP, *p*-NPPC, and BNPP in water; kinetics of *p*-NP adsorption on pure PA6, and PA6-CeAMM, and PA6-CePER samples in water; dephosphorylation activity of PA6-CeAMM, PA6-CePER, and pure PA6 nanofibers towards *p*-NP-TMP, *p*-NPPC, and BNPP in acetonitrile; zeta potential of CeNPs and pristine and CeO_2_-modified PA6 nanofibers; DFT specific surface area (SSA) and porosity (*V*_pore_) of pristine and CeO_2_-modified PA6 nanofibers (PDF)
