# Supplementary material for: Electrospun PA6 Nanofibers Bearing the CeO2 Dephosphorylation Catalyst
Source: ACS Omega. 2023 Jul 12;8(29):26610–8. doi: 10.1021/acsomega.3c03561 (PMC10373190; doi:10.1021/acsomega.3c03561)
Supplement: Supplementary file 1 — ao3c03561_si_001.pdf [file ao3c03561_si_001.pdf]

SUPPORTING INFORMATION:

Electrospun PA6 nanofibers bearing CeO_2 dephosphorylation catalyst

Jiří Henych,[*†,‡] Petr Ryšánek,[§] Martin Šťastný,[†] Zuzana Němečková,[†] Slavomír Adamec,[‡] Martin Kormunda,[§] Simona Kamínková,[§] Kateřina Hamalová,[§] Jakub Tolasz,[†] and Pavel Janoš[‡]*

† Institute of Inorganic Chemistry of the Czech Academy of Sciences, 250 68 Husinec-Řež, Czechia

‡ Faculty of Environment, Jan Evangelista Purkyně University in Ústí nad Labem, Pasteurova 3632/15, 400 96 Ústí nad Labem, Czechia

§ Faculty of Science, Jan Evangelista Purkyně University in Ústí nad Labem, Pasteurova 3632/15, 400 96 Ústí nad Labem, Czechia

*Corresponding author e-mail address: henych@iic.cas.cz

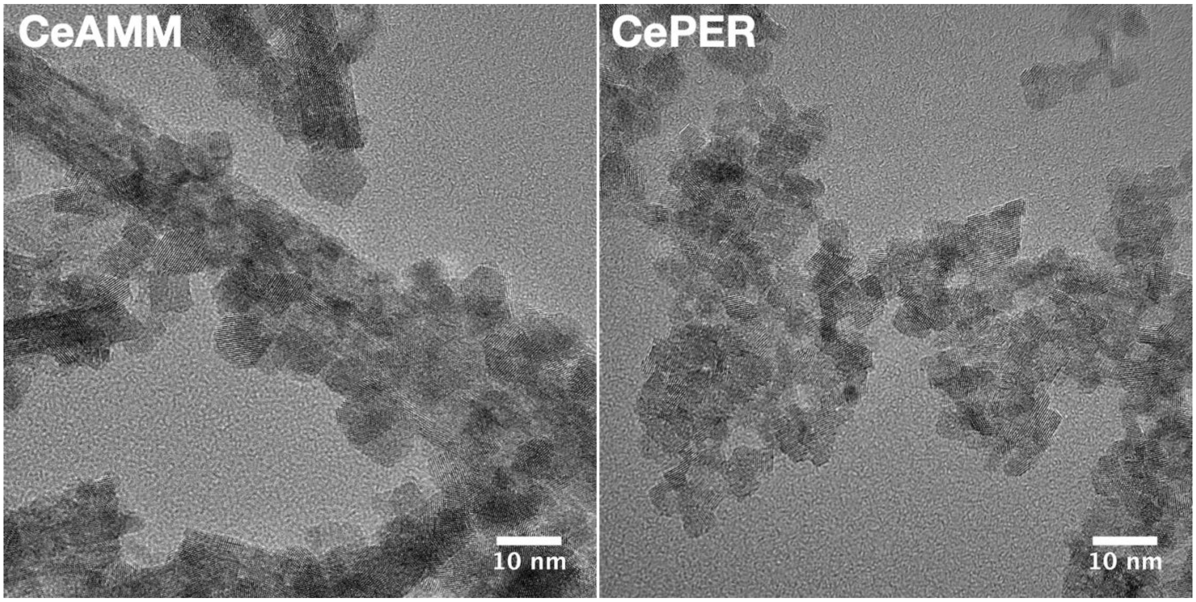

Figure S1. HRTEM images of CeNPs prepared by two different precipitation methods.

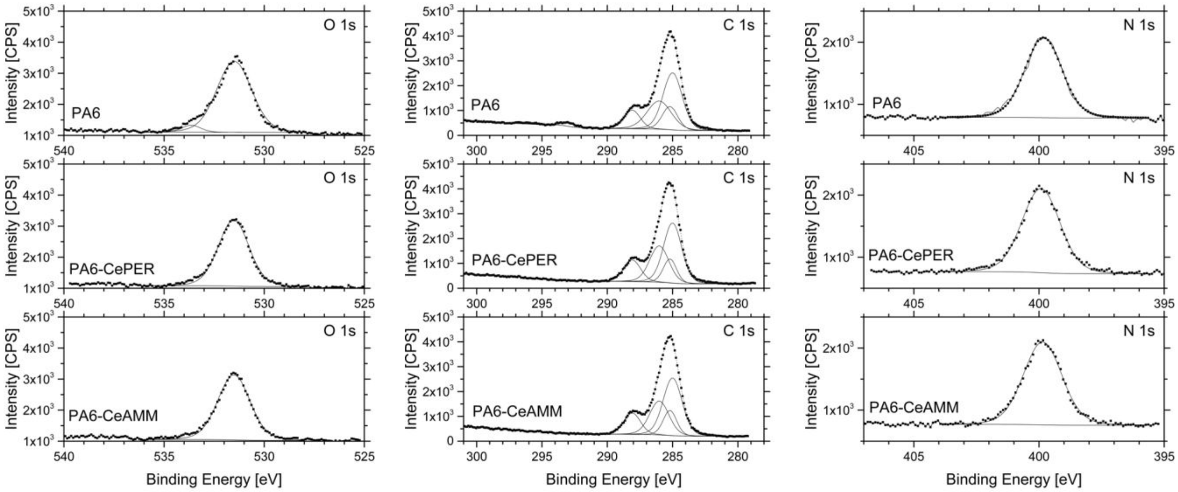

Figure S2. High-resolution XPS spectra of C 1s (left column), O 1s (middle column) and N 1s (right column) of pure PA6 nanofibers (top row), PA6-CePER (middle row) and PA6-CeAMM (bottom row)

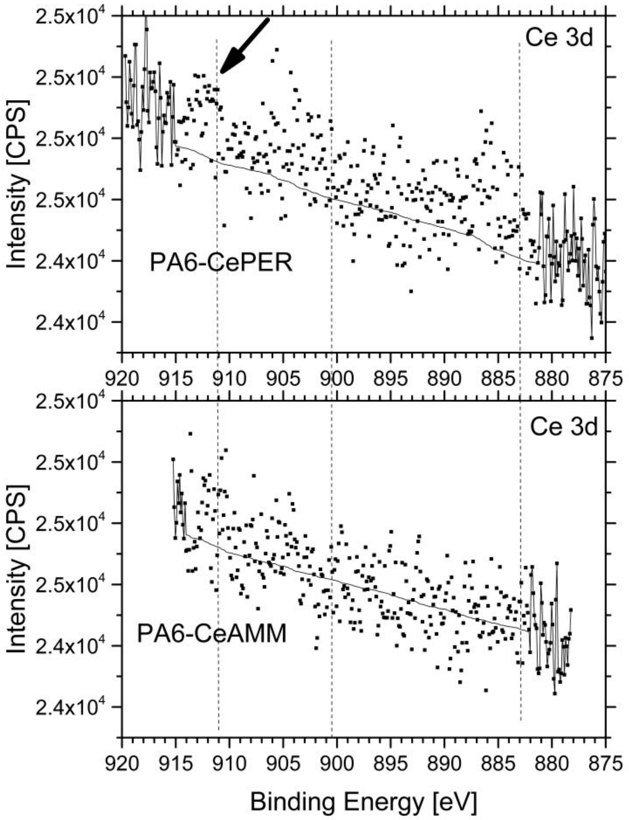

Figure S3. High-resolution XPS spectra of Ce 3d on PA6-CePER (top) and PA6-CeAMM (bottom)

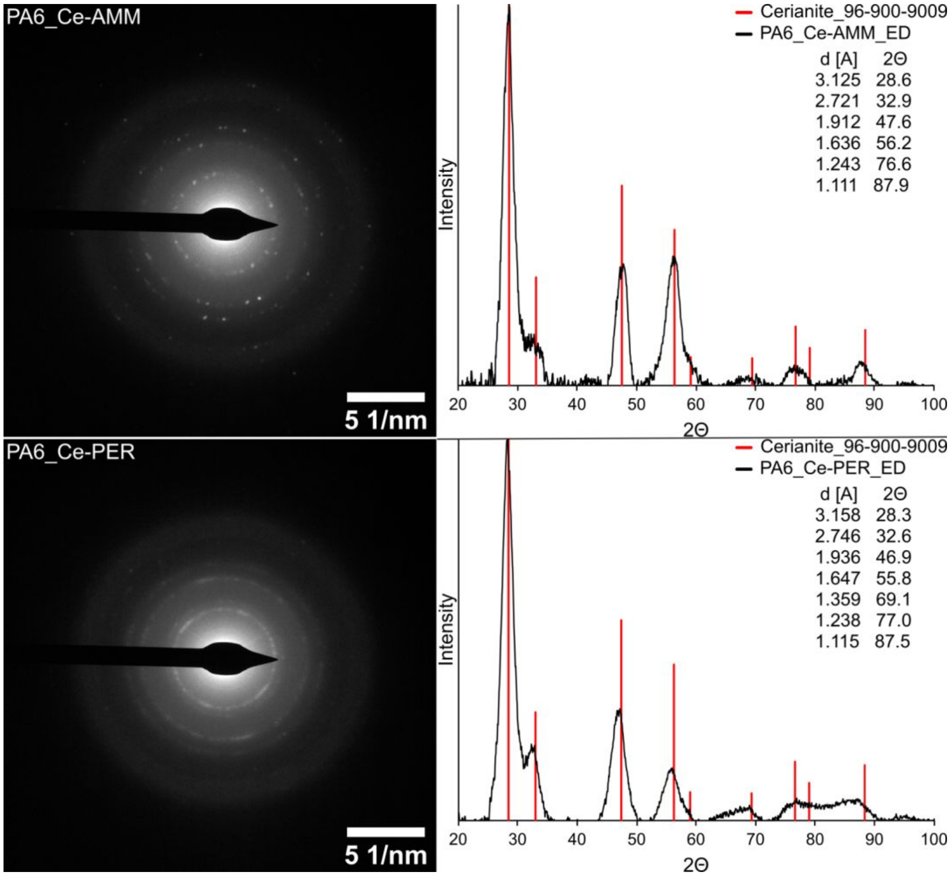

Figure S4. Electron diffraction analysis of the PA6-CeAMM and PA6-CePER samples.

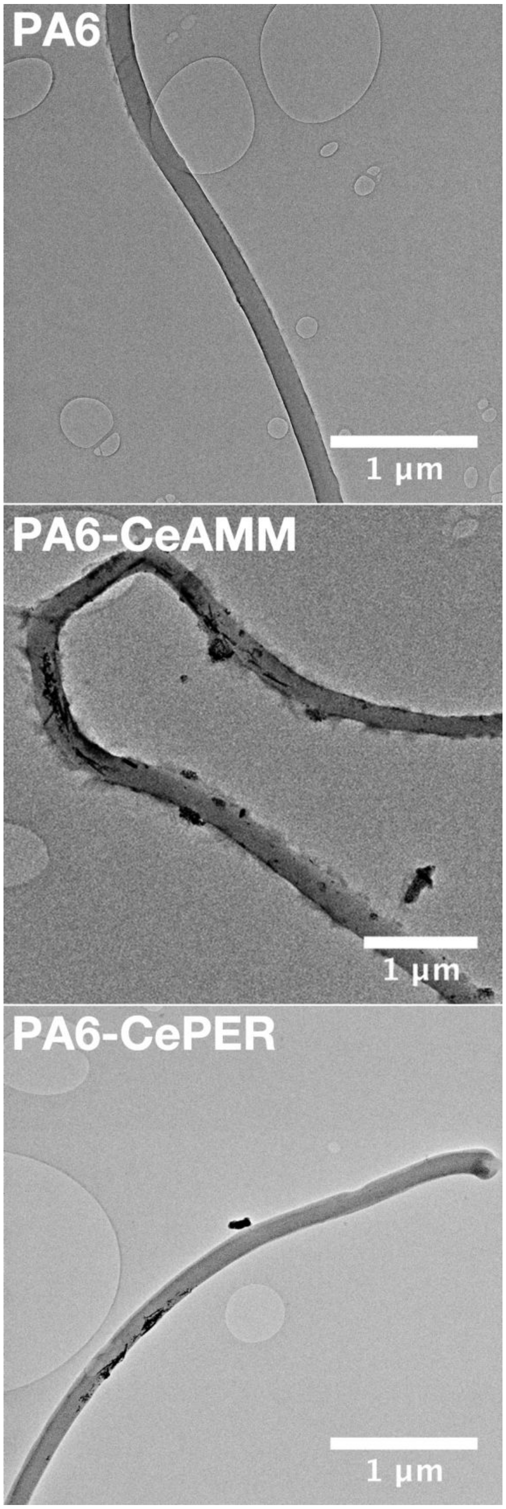

Figure S5. TEM images of pristine and CeO_2-modified PA6 nanofibers.

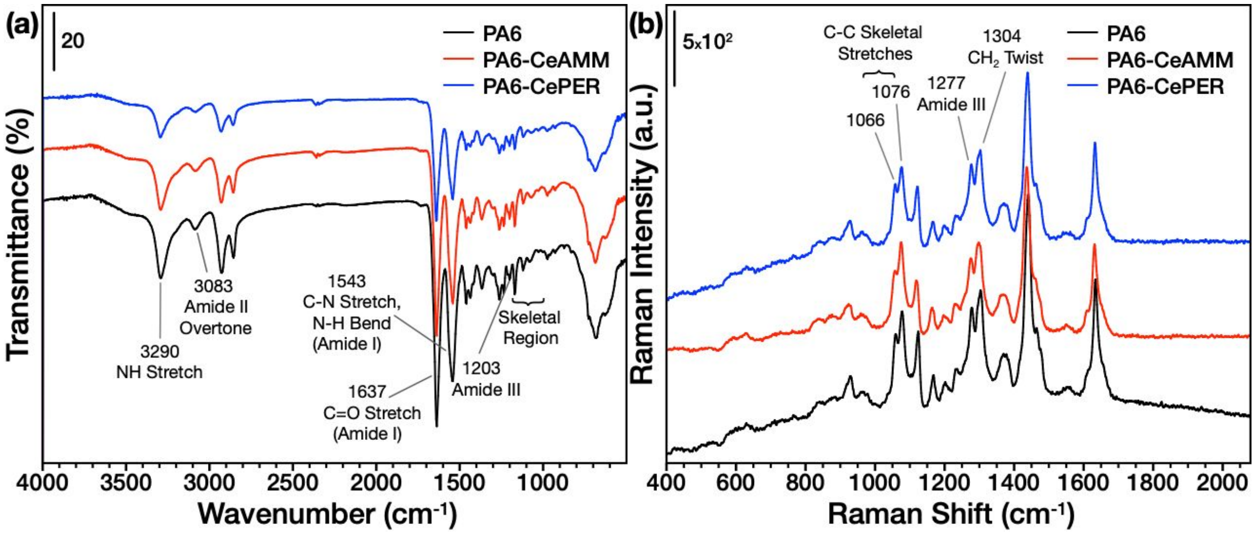

Figure S6. (a) FTIR and (b) Raman spectra of pristine and CeO_2-modified PA6 nanofibers

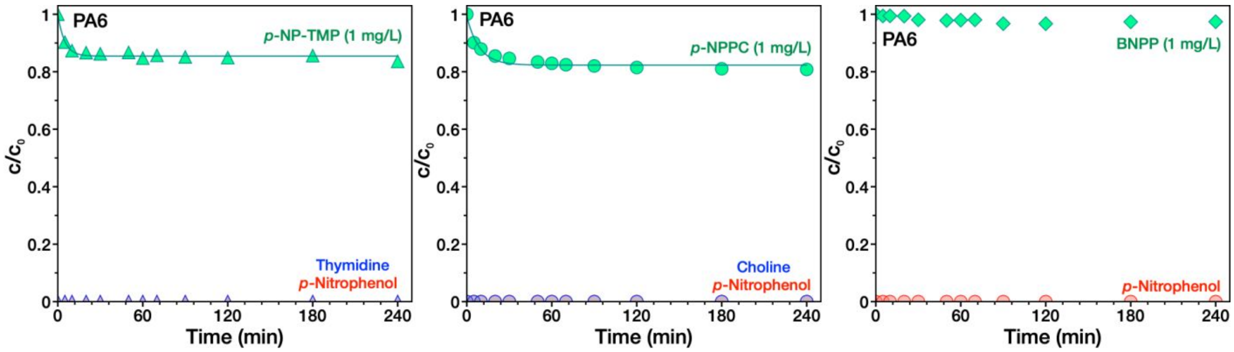

Figure S7. Dephosphorylation activity of pure PA6 nanofibers towards *p*-NP-TMP (left), *p*-NPPC (middle), and BNPP (right) in water.

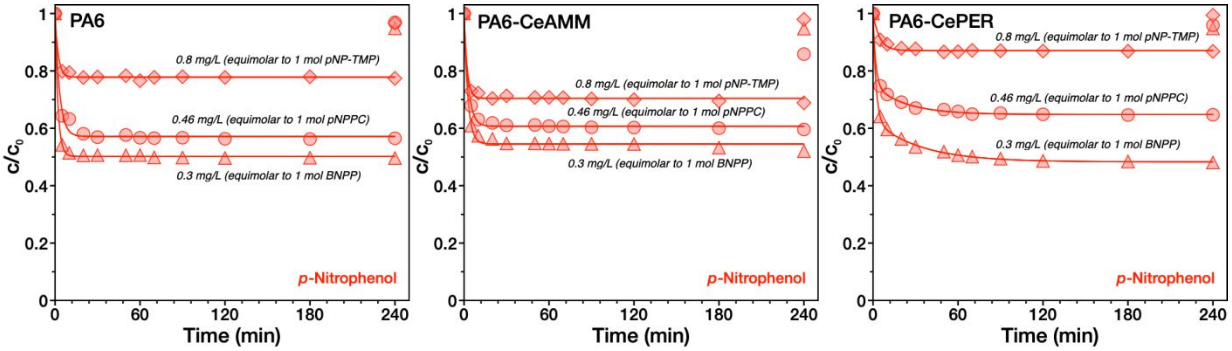

Figure S8. Kinetics of *p*-NP adsorption on pure PA6, and PA6-CeAMM, and PA6-CePER samples in water.

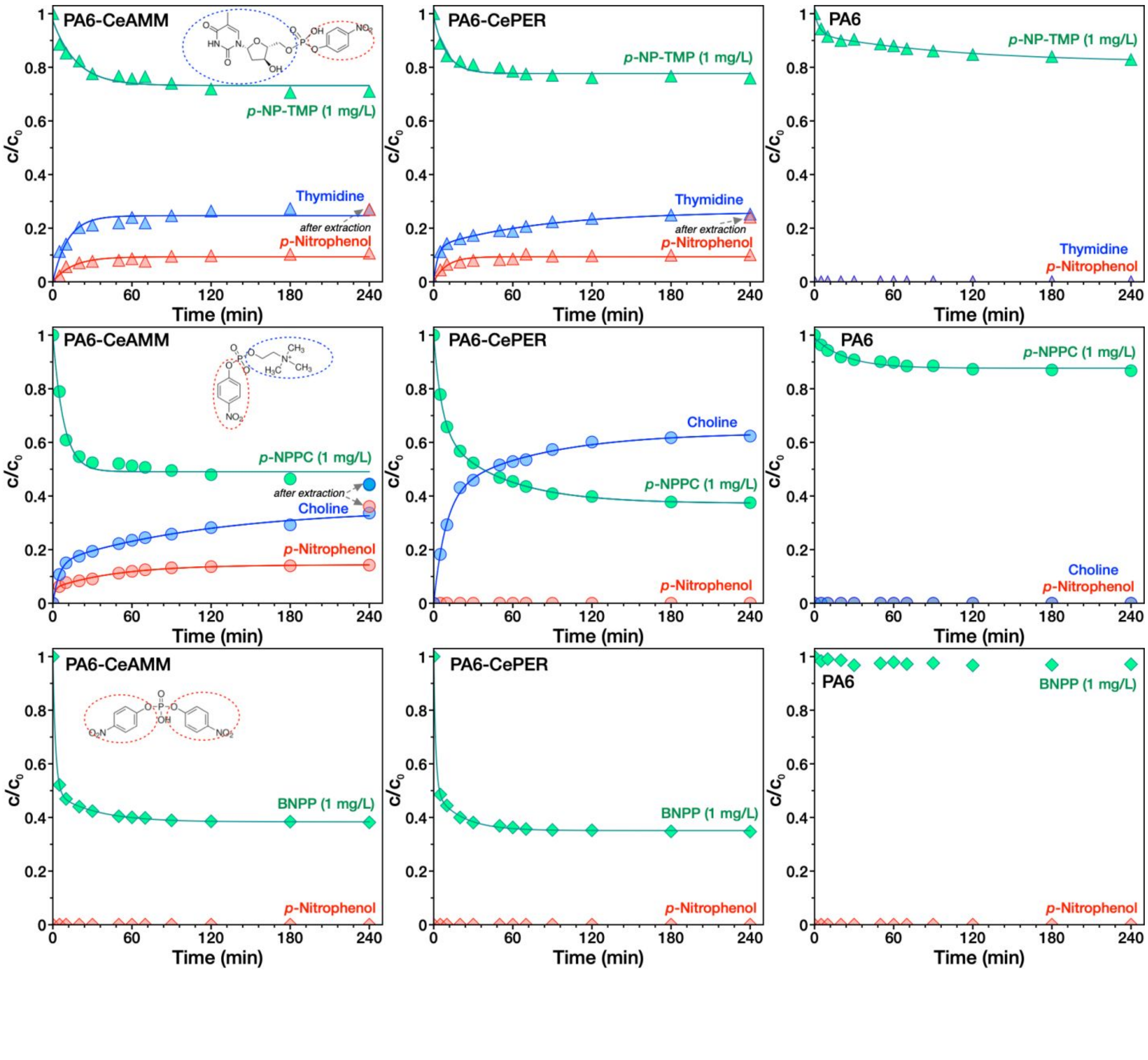

Figure S9. Dephosphorylation activity of PA6-CeAMM (left collumn), PA6-CePER (center column) and pure PA6 nanofibers (right column) towards *p*-NP-TMP (top line), *p*-NPPC (middle line), and BNPP (bottom line) in acetonitrile.

Table S1. Zeta potential of CeNPs and pristine and CeO_2-modified PA6 nanofibers.

Sample	Zeta potential, mV	SD
PA6	-37.40	0.60
PA6-CeAMM	-4.95	0.10
PA6-CePER	-9.41	1.61
CeAMM*	20.1	6.8
CePER*	30.6	7.6

*our previous work (Environ. Sci.: Nano, 2022,9, 3485-3501)

Table S2. DFT specific surface area (SSA) and porosity (V_{pore}) of pristine and CeO_2-modified PA6 nanofibers.

Sample	SSA, m^2/g	V_{pore}, cm^3/g
PA6	10.5±2.6	0.015±0.0004
PA6-CeAMM	11.4±0.2	0.020±0.002
PA6-CePER	7.0±0.5	0.020±0.001
